# TCR Repertoire as a Novel Indicator for Immune Monitoring and Prognosis Assessment of Patients With Cervical Cancer

**DOI:** 10.3389/fimmu.2018.02729

**Published:** 2018-11-22

**Authors:** Jin-Huan Cui, Kai-Rong Lin, Song-Hua Yuan, Ya-Bin Jin, Xiang-Ping Chen, Xi-Kang Su, Jun Jiang, Ying-Ming Pan, Shao-Long Mao, Xiao-Fan Mao, Wei Luo

**Affiliations:** ^1^Clinical Research Institute, The First People's Hospital of FoShan (Affiliated FoShan Hospital of Sun Yat-sen University), Foshan, China; ^2^Department of Gynecology, The First People's Hospital of FoShan (Affiliated FoShan Hospital of Sun Yat-sen University), Foshan, China; ^3^Department of Clinical Laboratory, The First People's Hospital of FoShan (Affiliated FoShan Hospital of Sun Yat-sen University), Foshan, China; ^4^Department of Abdominothoracic Radiotherapy, The First People's Hospital of FoShan (Affiliated FoShan Hospital of Sun Yat-sen University), Foshan, China

**Keywords:** cervical cancer, T cell receptor repertoire, biomarker, high-throughput sequencing, immune monitoring, prognosis

## Abstract

There is increasing evidence that deep sequencing-based T cell repertoire can sever as a biomarker of immune response in cancer patients; however, the characteristics of T cell repertoire including diversity and similarity, as well as its prognostic significance in patients with cervical cancer (CC) remain unknown. In this study, we applied a high throughput T cell receptor (TCR) sequencing method to characterize the T cell repertoires of peripheral blood samples from 25 CC patients, 30 cervical intraepithelial neoplasia (CIN) patients and 20 healthy women for understanding the immune alterations during the cervix carcinogenesis. In addition, we also explored the signatures of TCR repertoires in the cervical tumor tissues and paired sentinel lymph nodes from 16 CC patients and their potential value in predicting the prognosis of patients. Our results revealed that the diversity of circulating TCR repertoire gradually decreased during the cervix carcinogenesis and progression, but the circulating TCR repertoires in CC patients were more similar to CIN patients than healthy women. Interestingly, several clonotypes uniquely detected in CC patients tended to share similar CDR3 motifs, which differed from those observed in CIN patients. In addition, the TCR repertoire diversity in sentinel lymphatic nodes from CC patients was higher than in tumor tissues. More importantly, less clonotypes in TCR repertoire of sentinel lymphatic node was associated with the poor prognosis of the patients. Overall, our findings suggested that TCR repertoire might be a potential indicator of immune monitoring and a biomarker for predicting the prognosis of CC patients. Although functional studies of T cell populations are clearly required, this study have expanded our understanding of T cell immunity during the development of CC and provided an experimental basis for further studies on its pathogenesis and immunotherapy.

## Introduction

Cervical cancer (CC) is the fourth mostly commonly diagnosed cancer worldwide among women (1). The disease develops over many years through a series of precancerous lesions. The persistent infection of high-risk type human papilloma virus (HPV) is the primary cause of CC (2). In the development and progression of CC, the host immune status, particularly the immune state of T cells, plays an important role in the anti-tumor or anti-HPV infection mechanism (3, 4). Although there is a relatively larger amount of information on the role of immunotherapy and HPV vaccine strategies against CC (5, 6), only a few studies have explored the association between T cell immune response and the pathogenesis of CC, which is important for development of effective immunotherapy for CC patients.

Recently, a series of studies have characterized the signatures of T cell repertoires in patients with various types of cancer using high throughput T cell receptor (TCR) sequencing and confirmed that TCR repertoire could sever as a biomarker for monitoring immune response (7–9). However, the characteristics of TCR repertoire including diversity and similarity, as well as its prognostic significance in CC patients remain unknown. TCR is a specific molecule on the surface of T cell, responsible for recognizing antigens presented by the major histocompatibility complex (MHC) (10). In humans, most of TCRs consist of α and β chains (11). TCR β chain is generated by the random recombination of variable (V), diversity (D) and joining (J) gene segments, which generates the highly variable complementary determining region 3 (CDR3) that is critical for the specificity and affinity of antigen recognition (12). CDR3 polymorphisms account for TCR diversity and allow T cells to target any endogenous or exogenous antigen (13). T cells will be specifically activated and expanded through recognizing disease-associated antigens, which lead to a skewed distribution of TCR repertoire. Hence, the analysis of TCR repertoire can be used to reflect the immune responses for patients.

It has been reported that there were substantial differences in the TCR repertoire among women with HPV16-related cervical intraepithelial neoplasia (CIN) compared to women who cleared infection (14). However, the changes of TCR repertoire during cervical carcinogenesis and progression have not yet been described. In our study, we had performed the high-throughput TCR sequencing to characterize the TCR repertoire in peripheral blood samples from CC, CIN patients and healthy women in order to understand the property and alteration of T cell immunity in the process of the occurrence and development of CC. Moreover, we had also investigated the characteristics of the TCR repertoires in tumor tissues and paired sentinel lymph nodes from CC patients, as well as explored their potential value in the prognosis of CC.

## Materials and methods

### Sample collection

Peripheral blood samples were collected from a total of 25 CC patients, 30 CIN patients and 20 healthy women. Tumor tissue and paired sentinel lymph node specimens were collected from 16 CC patients. All of the participants recruited in this study were from the Affiliated Foshan Hospital of Sun Yat-Sen University (Guangdong, China) and this study was approved by the ethics committee of the Affiliated Foshan Hospital of Sun Yat-Sen University. None of them had received any chemotherapy or radiotherapy before sampling. All of the CC patients and CIN patients were proved by pathologic results. The tissue samples were dissected and harvested during surgery only when they were confirmed independently by three pathologists with extensive clinical experience. There were no lymph node metastases found in these CC patients undergoing surgery and all the sentinel lymph node specimens collected were normal lymph nodes. The clinical stages of the CC patients were differentiated according to the standards of the International Federation of Gynecology and Obstetrics (FIGO).

### RNA isolation and TCR sequencing

PBMCs were isolated from 10 ml fresh anticoagulant peripheral blood by density gradient centrifugation. All tissue and PBMC specimens were lysed with TRIzol® Reagent (Invitrogen, USA) and frozen at −80°C until further processing. Total RNA was extracted from 1 ml of tissue or PBMCs lysate using total RNA Kit (OMEGA, USA) according to the manufacturer's instructions.

TCRβ library for sequencing was constructed by a semi-nested PCR amplification and 5′ rapid amplification of cDNA ends (RACE) as previously described (15). First, about 1 μg of the total RNA was reverse-transcribed into the first-stand 5′ RACE-ready cDNA using SMARTer PCR cDNA synthesis kit (Clontech, USA) to add universal adapters at the 5′-end of the RNA and 3′-end of the cDNA. For the first round 5′ RACE PCR, 1 μg of a cDNA template was amplified with Nested Universal Primer (NUP, Clontech, 5′-AAGCAGTGGTATCAACGCAGAGT-3′) and 3′-TCR β constant region first round reverse primer (5′-AGATCTCTGCTTCTGATGGCT-3′) using Advantage 2 Polymerase mix (Clontech, USA). For the second round 5′ RACE PCR, first round agarose gel-purified PCR products were then amplified with NUP and 3′-TCR β constant region second round reverse primer (5′-TGGCTCAAACACAGCGACCT-3′). The two 3′-TCR β constant region reverse primers had been proved to be reliable and valid nested primers for TCR specific amplification in our previous studies (16–18). Finally, 1.5 μg agarose gel-purified library per sample was sequenced on the Hiseq 2500 platform.

### Sequencing data analysis

The raw data were stored in FASTQ format and the low-quality sequences were firstly filtered out according to four strict criteria: (1) contaminated by the adapter sequence; (2) with more than 5% uncalled bases (N); (3) with an average Phred-type Q-score < 15; and (4) PE reads with low-quality base readings (Q-score < 10) at the ends of reads. We then used BLAT (stepSize = 5 – minIdentity = 0 – minScore = 0) to align the sequence reads to TCR reference genes that were downloaded from IMGT/GeneDB database. The complete sequences that contain Variable (V) gene, Joining (J) gene and Constant (C) gene were translated into amino acid (aa) sequences, which were also defined as CDR3 aa sequences. The term unique aa sequences was used to describe the CDR3 aa sequences that comprised of a unique CDR3 aa sequence, which was also called clonotypes (19). CDR3 motifs were identified by clustering and comparing the total CDR3 aa sequences using the CD-HIT program (20). The motif models were constructed using Weblogo 3.0. The source code of our own TCR sequence analysis bioinformatical tool may be available on request, as well as the raw data.

The diversity of each sample was calculated by the Shannon's entropy (H) index, which took account both the sample richness and the degree of unevenness in the frequencies of CDR3 aa sequences (21). Entropy was calculated by summing the frequency of each clone times the log (base 2) of the same frequency over all productive reads in a sample (22). The higher the H index, the more diverse the CDR3 clones distribution (23). The similarity of the two different samples was assess using the TCR repertoire overlap, which was defined as the total number of sequencing reads from shared TCRβ sequences divided by the sum of sequencing reads detected in both samples, ranging from 0 to 1 (24).

### Statistical analysis

Comparisons between any two groups were analyzed using the Student's *t*-test, Paired *t*-test, Mann-Whitney U tests if appropriate. The differences of the measurement data between any two groups were firstly analyzed by the homogeneity test of variances. *T*-test was used when the homogeneity test of variances was not significant; otherwise Mann-Whitney *U*-test was used. Paired *t*-test was used to compare the paired samples. Two-sided *P*-values < 0.05 were considered statistically significant. *P*-value was corrected the by Benjamini–Hochberg Method when involving extensive multiple testing issues. These analyses were performed using Graphpad Prism software (version 5.1) and SPSS (version 20.0). Principal component analysis (PCA) was performed by R (version 3.4.3).

## Results

### The basic information of patients and sequencing data

We performed a high-throughput TCR sequencing on the peripheral blood samples from 25 CC patients, 30 CIN patients and 20 healthy women, as well as on the tumor tissue and paired sentinel lymph node samples from another 16 CC patients, and analyzed their TCR sequencing data. The clinical characteristics of the CC patients were listed in Tables 1; Tables S1, S2. Among patients who were collected peripheral blood samples, there were 11 (40.0%) patients with early CC (stage IB1) and 14 (60%) patients with advance CC (stage IB2-IVB). The 16 patients who were collected tissue samples were almost early CC patients. Among them, except for one patient lost to follow-up, 8 (50%) patients were alive with disease non-progression and seven (43.75%) patient were alive with disease progression at last follow-up after surgery.

**Table 1:** Clinical characteristics of patients with CC.

**Clinical characteristics**	**PB (*N* = 25)**	**T&LN (*N* = 16)**
***N* = 41**	***N* (%)**	***N* (%)**
**AGE (YEARS)**		
≤ 50	15 (60.0)	10 (62.5)
>50	10 (40.0)	6 (37.5)
**FIGO STAGE**		
IB1	11 (44.0)	14 (87.5)
IB2	4 (16.0)	2 (12.5)
IIA	1 (4.0)	/
IIB	3 (12.0)	/
IIIB	3 (12.0)	/
IVA	1 (4.0)	/
IVB	2 (8.0)	/
**PATHOLOGICAL TYPE**		
Squamous cell carcinoma	18 (72.0%)	15 (93.75)
Adenocarcinoma	6 (24.0%)	/
Adenosquamous carcinomas	1 (4.0%)	1 (6.25)
**PATHOLOGICAL GRADING**		
Moderately differentiated	22 (88.0%)	13 (81.25)
Moderately and poorly differentiated	2 (8.0%)	1 (6.25)
Poorly differentiated	1 (16.0%)	2 (12.5)
**DISEASE STATUS AT LAST FOLLOW-UP**		
Disease-prognosis	/	7 (43.75)
Non-prognosis	/	8 (50.0)
Unknown	/	1 (6.25)

*PB, peripheral blood samples; T, tumor tissue samples; LN, sentinel lymph node samples*.

*The disease status of patients who were collected peripheral blood samples has no sense of statistical analysis due to the short follow-up times*.

*Disease-prognosis indicates patients were alive with tumor recurrence or metastasis or had died of their disease after surgery; Non-prognosis indicates patients were alive with stable disease after surgery at last follow-up; Unknown indicates patients lost to follow-up*.

Profiling the sequencing data from 75 blood samples, a total of 94,091,782 TCRβ CDR3 aa sequences were obtained, with an average of 1,254,557 per sample. All the 65 distinct V and 13 distinct J genes were observed, with a median number of 63 V genes (ranging from 55 to 65; Table S1). In addition, a total of 45,614,805 TCRβ CDR3 aa sequences were obtained from 16 pairs of tissue samples, with an average of 1,425,462 per sample. A median number of 62 V genes (ranging from 58 to 64) and 13 J genes were identified in each sample (Table S2).

### TCR repertoire diversity in peripheral blood samples from CC, CIN patients, and healthy women

**Figure 1:**
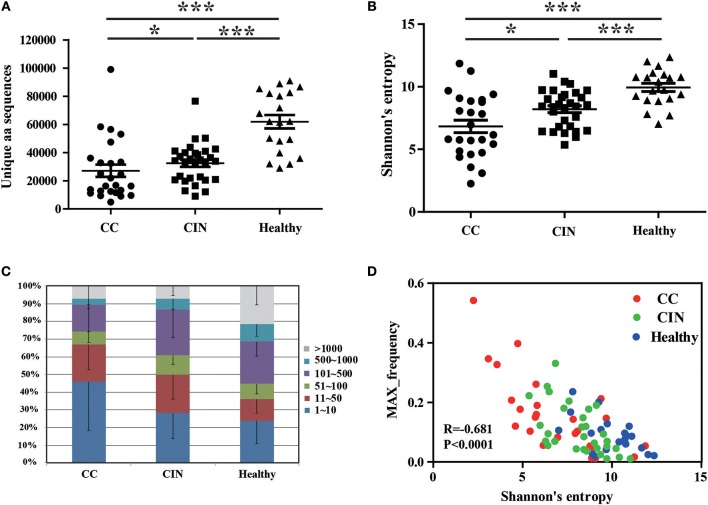
Clonal distributions of peripheral blood T cell repertoires in the CC, CIN patients and healthy women. **(A)** The number of unique TCR CDR3 aa sequences and **(B)** The Shannon's entropy of TCR repertoire in peripheral blood of the CC, CIN patients and Healthy women. Bars depict the mean (±SEM) of the groups. ^*^P < 0.05, ^***^P < 0.001, Kruskal wallis test. **(C)** Percentage of the top 1,000 frequent TCR CDR3 aa sequences in each group. **(D)** The correlation between the Shannon's entropy and the frequency of the largest dominant clone, Pearson correlation test.

We firstly calculated the number of the TCRβ CDR3 unique aa sequences and the Shannon's entropy (H) index to evaluate the diversity of TCR repertoire in each peripheral blood sample, and then compared them among the CC, CIN and healthy groups. The results showed that the number of the unique aa sequences in CC patients (27,081 ± 4,358, mean ± standard error of the mean [SEM]) was significantly lower than CIN patients (32,437 ± 2,537, *P* = 0.043) and healthy women (61,878 ± 4,750, *P* < 0.001). The number of the unique aa sequences in CIN patients was significantly lower than in healthy women (*P* < 0.001; Figure 1A). Similarly, there was a significant decrease of the H diversity index in CC patients (6.830 ± 0.5045) compared with CIN patients (8.209 ± 0.2719, *P* = 0.018) and healthy women (9.943 ± 0.3260, *P* < 0.001). The H diversity index in CIN patients was also significantly lower than in healthy women (*P* < 0.001; Figure 1B). These result data indicated that T cell repertoire diversity was lowest in CC patients, followed by CIN patients, and highest healthy women, suggesting that the circulating T cell repertoire diversity gradually decrease during cervical carcinogenesis.

The clonal expansion of TCR repertoire was further assessed by calculating the frequency distribution of the CDR3 aa sequences. We found that the proportion of the top 10 clonotypes was higher in CC patients than CIN patients and healthy women, while more low-frequency sequences were observed in healthy women (Figure 1C). In addition, there was an inverse correlation between the H diversity index of TCR repertoire and the frequency of the largest dominant clone (*R* = −0.681, *P* < 0.0001; Figure 1D). Taken together these data suggested that the decrease of TCR repertoire diversity can be reflected by the emergence of the highly expanded clones.

### Relationship between the peripheral blood TCR repertoire diversity and clinical characteristics in CC patients

**Figure 2:**
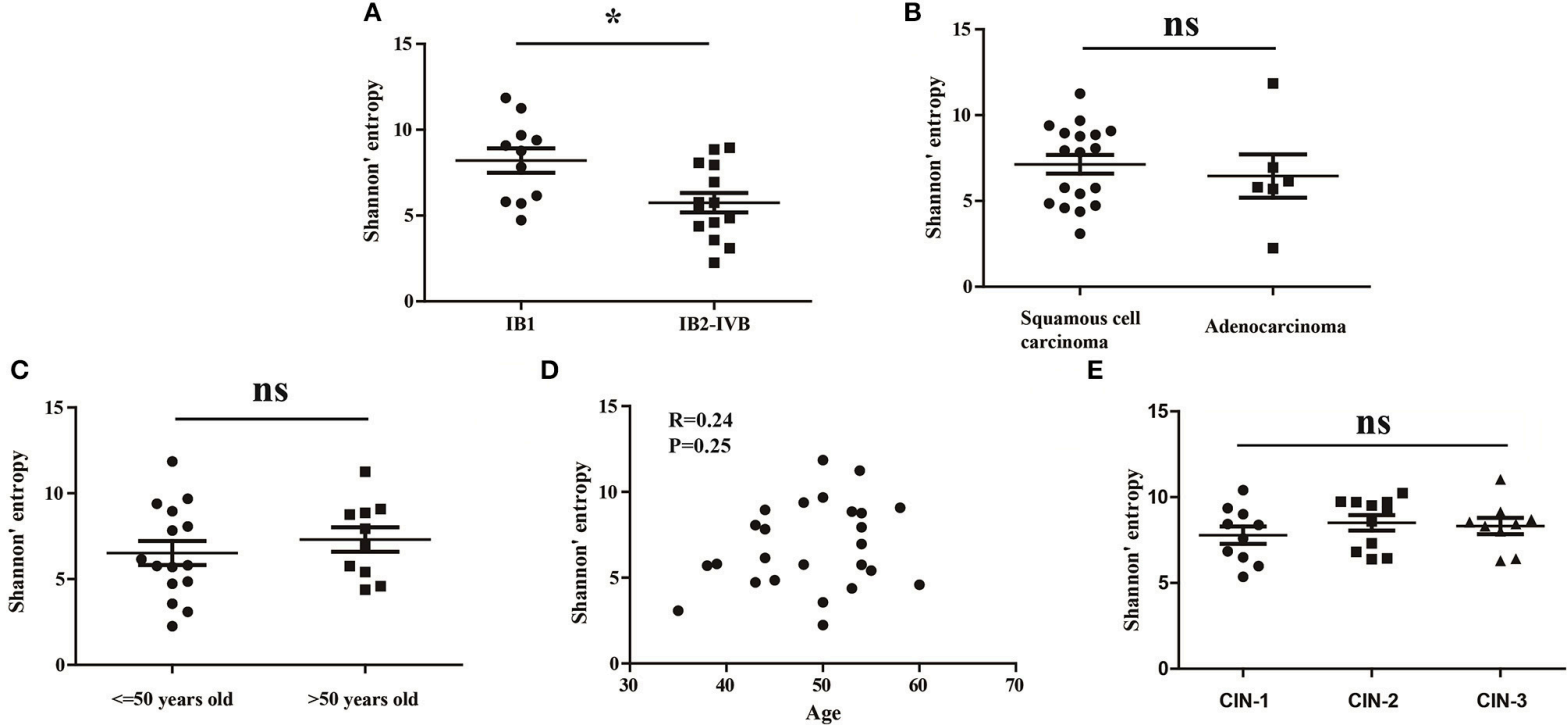
The Shannon's entropy of peripheral blood TCR repertoire between different subgroups in CC or CIN patients. Comparison of the Shannon's entropy **(A)** between the early and advance CC patient subgroups, **(B)** between the squamous cell carcinoma and adenocarcinoma subgroups, and **(C)** between age > 50 and age ≤ 50 years old subgroups for CC patients. *P < 0.05, ns, no significant difference, *t*-test. **(D)** The correlation between age and Shannon's entropy in CC patients, Pearson correlation test. **(E)** Comparison of the Shannon's entropy in peripheral blood TCR repertoire among CIN1, CIN2, and CIN3 subgroups. ns, no significant difference, one-way ANOVA test.

To investigate whether the clinical stage, pathological type or age of CC patients would influence the diversity of peripheral blood TCR repertoire, we performed the difference analysis between the distinct subgroups. Interestingly, our results showed that the TCR repertoire diversity in advance CC patients (5.521 ± 0.5508) were significantly lower than in early CC patients (7.566 ± 0.6670, *P* = 0.012; Figure 2A), suggesting that the peripheral blood TCR repertoire diversity decrease with the progression of CC patients. This also supported the conclusion that the circulating T cell repertoire diversity gradually decrease during cervical carcinogenesis. However, no significant differences of T cell repertoire diversity were observed between the squamous cell carcinoma and adenocarcinoma subgroups (Figure 2B) or between the different age subgroups (Figures 2C,D).

In addition, we also compared the differences of the TCR repertoire diversity among patients with distinct degrees of CIN. The results showed that no significant differences were observed among CIN-I, CIN-II, and CIN-III subgroups (Figure 1E), indicating that the diversity of peripheral blood T cell repertoire could not distinguish the different degrees of lesions in CIN patients.

### V and J gene usages of peripheral blood TCR repertoire in CC, CIN patients, and healthy women

**Figure 3:**
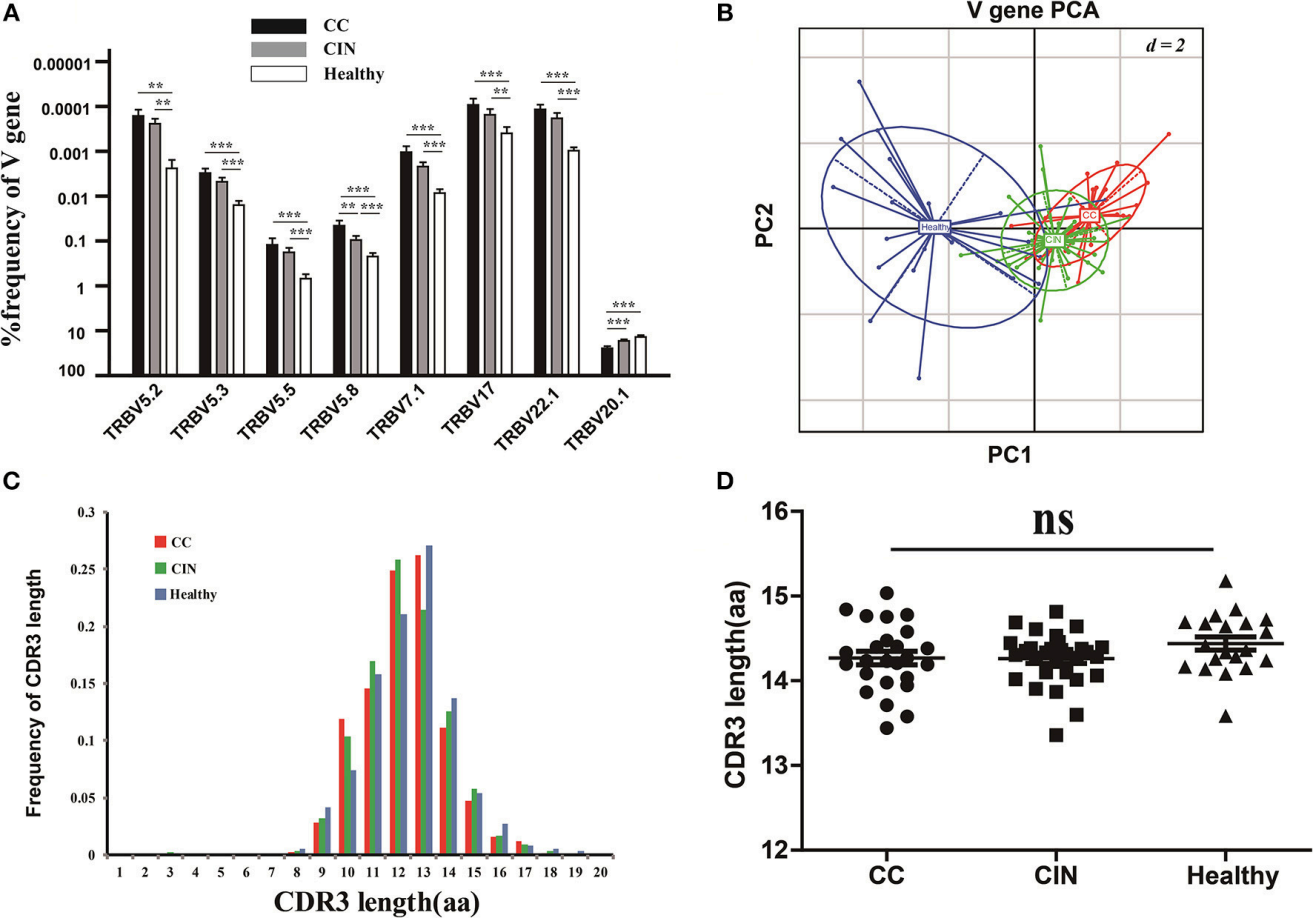
Comparisons of the TCRβ (TRB) V gene usages and the CDR3 length distributions in peripheral blood TCR repertoires among the CC, CIN and healthy groups. **(A)** The TRBV genes with usage differences among the CC, CIN, and Healthy groups. Bars depict the mean (±SEM) of each group. **P < 0.01, ***P < 0.001, one-way ANOVA test. **(B)** Principal component analysis of the TRBV genes with usage differences. PC1 refers to the first principal component, whereas PC2 indicates the second principal component. **(C)** The distribution of CDR3 lengths in the CC, CIN and healthy groups. **(D)** The CDR3 average length in the CC, CIN, and healthy groups. ns, no significant difference, one-way ANOVA test.

The V and J gene usage of each patient from the three groups were showed in Figure S1A. There were 8 out of 65 V gene usages with significantly differences among the three groups (Figure 3A). Of note, except for V5.8 and V20.1, the usage frequencies of the remaining six V genes were significantly higher in healthy women than CC and CIN patients, but not significantly different between CC and CIN patients. The results from the principal component analysis (PCA) also showed that the healthy samples could be clearly separated from the CC and CIN samples using these V gene usages, whereas the CC and CIN samples were partially overlapped (Figure 3B). These results suggested that the V gene usages in CC and CIN patients were more similar.

Moreover, we also compared the J gene usages and the length distribution of TCRβ CDR3 aa sequences among the CC, CIN and healthy groups. There were none of the J genes with usage differences among the groups. The CDR3 length distributions were similar among the groups, with no significant differences of the CDR3 average lengths (Figures 3C,D).

### Similarity of peripheral blood TCR repertoires within groups or between groups

**Figure 4:**
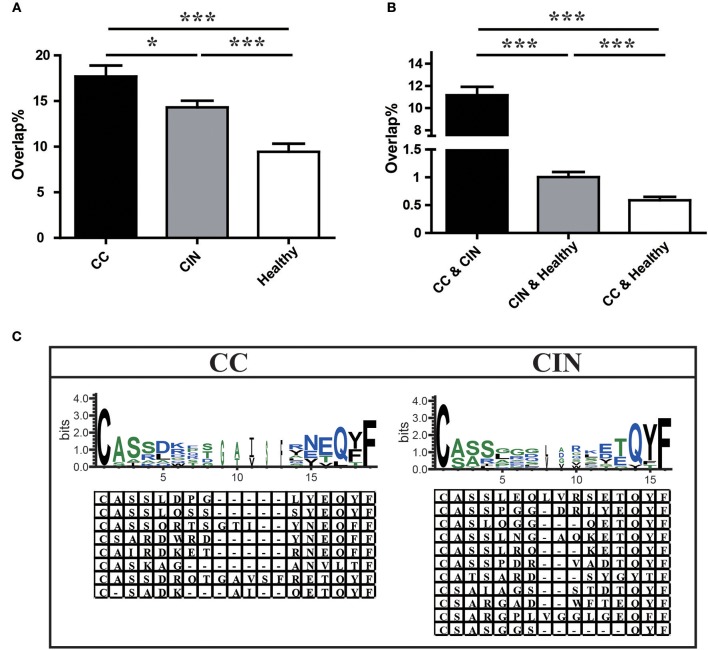
Comparison of clonotypes in peripheral blood TCR repertoire within each group and between different groups. **(A)** The overlap of clonotypes within each group, CC, CIN, and Health. **(B)** The overlap of clonotypes between every two groups, CC and CIN, CC and Health, and CIN and Health. *P < 0.05, ***P < 0.001, one-way ANOVA test. **(C)** The amino acid characteristics of disease-associated clonotypes in the CC (left) and CIN (right) patients, which were identified based on similarities in their CDR3 aa sequences using the CD-HIT program (90% quantile). Hydrophilic amino acids (RKDENQ) are shown in blue, neutral amino acids (SGHTAP) are shown in green, hydrophobic amino acids (YVMCLFIW) are shown in black.

We further assessed the TCR repertoire similarity by calculating the TCR repertoire overlap, both between samples within the same group and between the three different groups. We found that the average overlap between any two CC patients (17.69% ± 1.19%) was significantly higher than that seen in CIN patients (14.30 ± 0.73%, *P* = 0.024) and healthy women (9.44 ± 0.88%, *P* < 0.001). The difference in average overlap between the CIN and healthy groups was also statistically significant (*P* < 0.001; Figure 4A). More interestingly, the average overlap between CC and CIN patients (11.16 ± 0.75%) was significantly higher than that observed between CC patients and healthy women (0.59 ± 0.06%, *P* < 0.001) and between CIN patients and healthy women (1.01 ± 0.09%, *P* < 0.001; Figure 4B). These results indicated that the overlap in TCR repertoire within group was larger than that between different groups, and the peripheral blood TCR repertoires in CC patients more closely resembled those in CIN patients than healthy women.

### The disease-associated clonotypes detected in CC or CIN patients

We searched for clonotypes exclusively present in CC patients but absent from CIN patients and healthy women to identify the immune response characteristics specific to CC, which were defined as disease-associated clonotypes of CC patients. We found that those disease-associated clonotypes exclusively present in at least three CC patients were statistically significant using Fisher's exact test. A total of 887 disease-associated clonotypes of CC patients were identified, and 8 of them were found in more than 30% of CC patients (Figure 4C; Table S3). The former four clonotypes were found in 9 of 25 (36%) CC patients, while the latter four clonotypes were found in 8 of 25 (32%) CC patients. Similarly, a total of 1,328 disease-associated clonotypes of CIN patients that present in at least four CIN patients but not found in CC patients and healthy women (Fisher's exact test, *P* < 0.05) were identified, 11 of which were found in more than 30% of cases (Figure 4C; Table S3). The clonotype CASSLEQLVRSETQYF and the clonotype CASSPGGDRLYEQYF were respectively found in 11 of 30 (36.7%) and 10 of 30 (33.3%) CIN patients. The remaining nine clonotypes were found in 9 of 30 (30%) CIN patients.

Through observing the aa characteristics of these several disease-associated clonotypes, we found that a aspartic acid (D) at positions five and six of the CDR3 region emerged in 62.5% of the eight CC-associated clonotypes, while a hydrophobic residue glycine (G) at positions five to seven of the CDR3 region emerged in 63.6% of the 11 CIN-associated clonotypes. These results suggested that the disease-associated clonotypes of CC patients shared similar CDR3 motifs that differed from those observed in CIN patients.

### TCR repertoire diversity in tumor tissues and paired sentinel lymph nodes from CC patients

**Figure 5:**
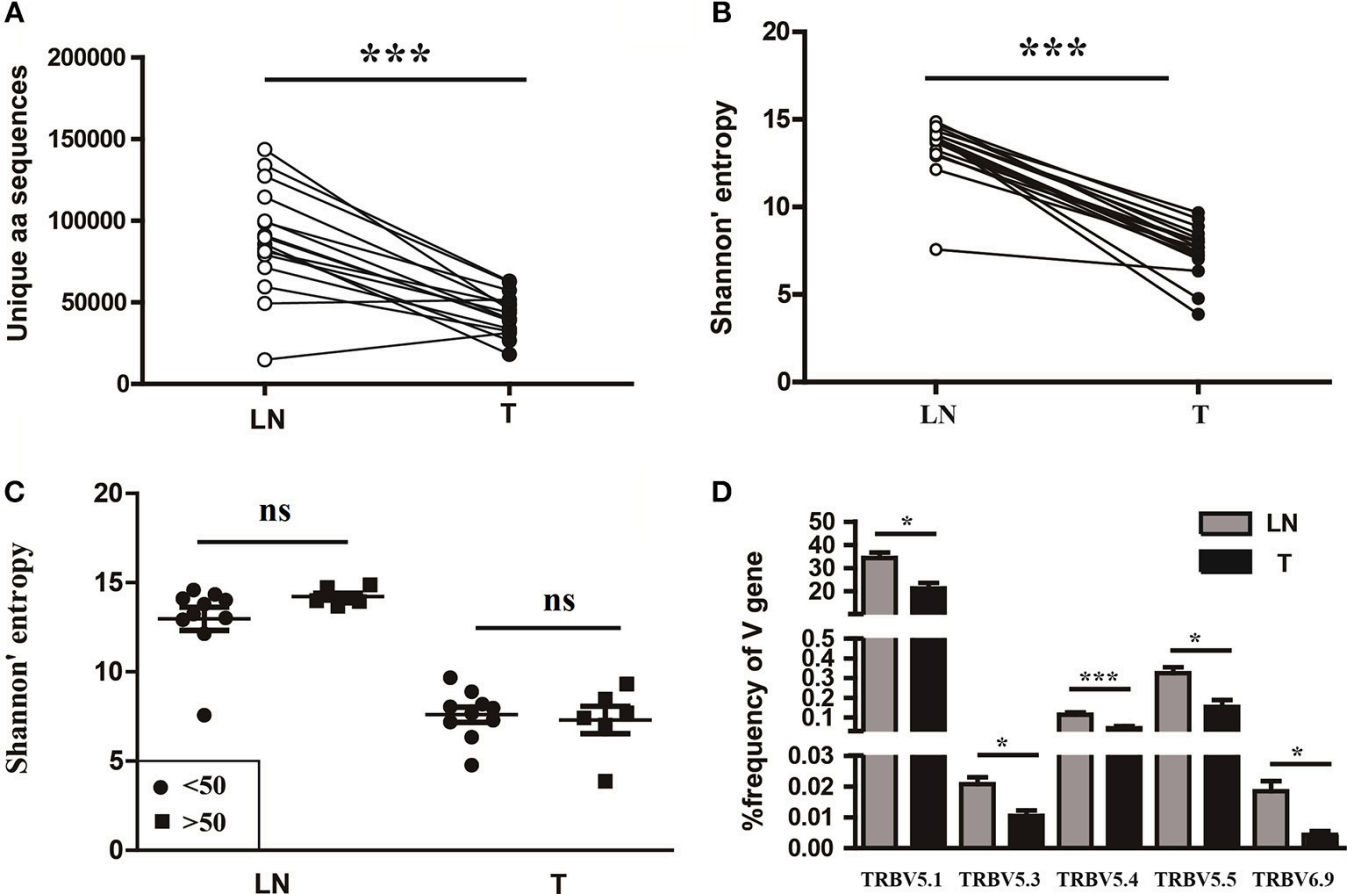
Variance analysis. Comparison of **(A)** the number of the unique aa sequences, **(B)** the Shannon's entropy of TCR repertoire between tumor tissues (T) and paired sentinel lymph nodes (LN) from CC patients. ***P < 0.001, paired *t*-test. **(C)** Comparison of the Shannon's entropy of TCR repertoire in tumor tissues (T) and sentinel lymph nodes (LN) between the age < 50 and age > 50 years old subgroups. ns, no significant difference, *t*-test. **(D)** The TCRβ (TRB) V genes with usage differences between T and LN groups. *P < 0.05, ***P < 0.001, paired *t*-test.

To understand the T cell clonal expression in tissue samples from CC patients, we compared the TCR repertoire diversity between tumor tissues and paired sentinel lymph nodes using the number of the TCRβ CDR3 unique aa sequences and the H diversity index. The results showed that both the number of the TCRβ CDR3 unique aa sequences and the H diversity index were significantly lower in tumor tissues than paired sentinel lymph nodes (*P* < 0.001; Figures 5A,B). Of note, there were also no influences of age on TCR repertoire diversity either in tumor tissues or in sentinel lymph nodes (Figure 5C).

In addition, we compared the differences of V and J gene usages between tumor tissues and sentinel lymph nodes (Figure S1B). The results showed that the V5.1, V5.3, V5.4, V5.5, and V6.9 gene usages were significant higher in sentinel lymph nodes than tumor tissues (Figure 5D), which implied a unique V gene usage pattern for TCR in tumor tissue.

### Association between TCR repertoire in tumor tissues or sentinel lymph nodes and the prognosis of CC patients

**Figure 6:**
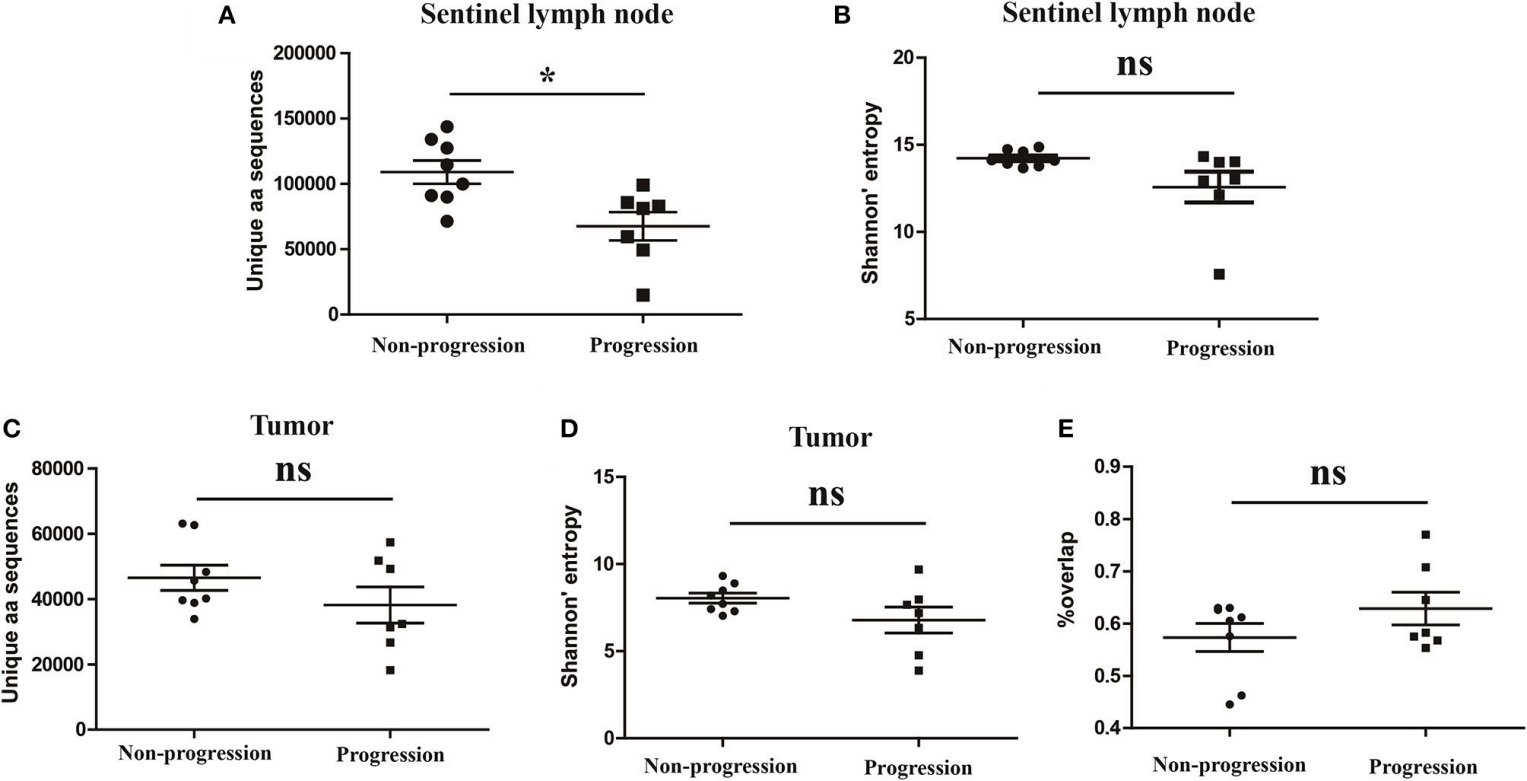
Variance analysis. Comparison of **(A)** the number of the unique aa sequences and **(B)** Shannon's entropy of TCR repertoire in sentinel lymph nodes between CC patients with disease progression and non-progression groups. Comparison of **(C)** the number of unique aa sequences and **(D)** the Shannon's entropy of TCR repertoire in tumor tissues between the progression and non-progression groups. **(E)** Comparison of the overlap of TCR repertoires of paired tissue samples between the progression and non-progression groups. Bars depict the mean (±SEM) of each group. *P < 0.05, ns, no significant difference, *t*-test.

It has been reported that TCR repertoire has great prognostic value in other types of cancers (25, 26), however, whether the TCR repertoire could predict the prognosis of CC patients remains unknown. We therefore attempted to explore the relationship of the TCR repertoire in tumor tissue or sentinel lymph node with the prognosis of the CC patients. The number of the unique aa sequences and the H diversity index in tumor tissues or sentinel lymph nodes, as well as the TCR repertoire overlap between tumor tissues and paired sentinel lymph nodes were compared among patients with different disease status after surgery. The results showed that the number of the unique aa sequences in sentinel lymph nodes from patients with disease-progression (67,560 ± 10,820) were significantly lower than those from patients with non-progression (109,000 ± 8,848, *P* = 0.0104, Figure 6A). That was to say, less clonotypes in the TCR repertoire of sentinel lymph node was associated with the poor prognosis of CC patients.

However, the number of the unique aa sequences in tumor tissues, the H diversity index in tumor tissues or sentinel lymph nodes and the TCR repertoire similarity between tumor tissues and paired sentinel lymph nodes did not show any significant differences between the patients with disease-progression group and the patients with non-progression group (Figures 6B–E).

## Discussion

The present study, to the best of our knowledge, is the first to describe the characteristics of T cell repertoire in the peripheral blood, tumor tissue and paired sentinel lymphatic node from CC patients using high-throughput TCR sequencing. With the help of our CIN and healthy blood sample sets, we found that the diversity of circulating TCR repertoire gradually decreased during the occurrence and development of CC, but the circulating TCR repertoires in CC patients were more similar to CIN patients than healthy women. Moreover, several clonotypes were uniquely detected in CC patients and they tended to share similar CDR3 motifs that differed from those observed in CIN patients. In addition, we found that the TCR repertoire diversity in normal sentinel lymphatic nodes from CC patients was higher than in tumor tissues. Interestingly, the number of clonotypes detected in normal sentinel lymphatic nodes was associated with the prognosis of the patients, the less clonotypes the poor prognosis

There is increasing evidence that the TCR repertoire diversity might serve as a potential biomarker of antitumor adaptive immunity and a predictive marker of responses to cancer therapy (27). Most previous studies focused on profiling the TCR repertoire in tumor-infiltrating lymphocytes (TILs) to investigate their correlation with clinical prognosis and seek tumor-specific T-cell clones for use in cancer immunotherapy (28, 29). However, it has also been reported that the signatures of TCR repertoire in peripheral blood could represent an imprint of antigenic repertoire present in various disease states, which could distinguish patients with different diseases (30), as well as could reflect the clinical response to immunotherapy in cancer patients (31, 32). In our study, we found that the circulating TCR repertoire diversity in CC patients was lowest, followed by CIN patients, with healthy women highest. In addition, we also found that the circulating TCR repertoire diversity in advance CC patients was significantly lower than that in early CC patients. These results indicated that the circulating T cell repertoire diversity gradually decrease during cervical carcinogenesis and progression, which could serve as a potential biomarker for monitoring the immune alterations of CC patients.

The oligoclonal T cell repertoire may signify failed selection of clonotypes that caused by lower TCR affinity, inappropriate antigen selectivity, poor antigen-presenting capacity, or T cell exhaustion and eventual disappearance over time (33). As demonstrated by studies of T cell function in chronic viral infections and malignant tumors, chronic antigen exposure leads to T cell exhaustion, generally characterized by up-regulated expression of inhibitory receptors and reduced ability to secrete effector cytokines (34). Both CIN and CC are indeed closely associated with HPV chronic infection, and peripheral T cell immune exhaustion induced by the PD-1 signaling pathway has also been observed in CIN and CC patients (35). T cell exhaustion might be the major reason for the decrease diversity of T cell repertoire in CIN and CC patients. Our findings that the circulating TCR repertoire diversity gradually decreased during the cervix carcinogenesis and progression might indicate the increasing degree of T cell exhaustion in this process. In addition, we also found that the highly expanded clones accounted for a larger proportion of TCR repertoire in CC patients relative to CIN patient and health women through analyzing the clonal frequency distribution of TCR repertoire. Although the function of these highly expanded clones had not been confirmed, this finding could roughly reflect why a low TCR repertoire diversity presented in CC patients because the low diversity theoretically means less clonotypes and (or) few dominated clonotypes in a TCR repertoire (21).

The V and J genes are the main components of the TCR repertoire. All the 65 distinct V and 13 distinct J genes were observed in our study. Our data revealed J gene usage patterns were similar among the CC, CIN and healthy groups, while several V gene usages were similar between the CC and CIN groups but significantly different from the healthy group. We further investigated the similarity of CDR3 aa sequences of TCR repertoire and found that a higher overlap between CC and CIN than between either of these and healthy women. The high similarity of TCR repertoire between CC and CIN patients might be attributable to HPV long-time infection, which would cause inflammation in cervix. Generally, years and decades are needed for HPV infection to advance to CIN and CC, thus CIN and CC patients might have similar immune environment, such as the same antigenic triggers.

Studies in multiple sclerosis (MS) have reported that the shared clonotypes that identified among MS patients show CDR3 sequence relatedness (consensus motifs), which could distinguish MS patients from patients with idiopathic intracranial hypertension (IIH) (36). Moreover, studies in three live diseases have also indicated that the disease-associated clonotypes shared similar TCRβ protein characteristics (19). Analogously, in our study several clonotypes uniquely identified in CC were observed to share similar CDR3 motifs that differed from those observed in CIN patients, although the antigen specificity of these clones remained unknown. Further studies are needed to determine whether this motif constitutes a CC-specific or CIN-specific signature and potential biomarker of disease in peripheral blood TCR repertoire.

There is a pity that the correlation between the circulating TCR repertoire and the CC prognosis had not been explored in our study due to the short follow-up times (averaging < 10 months). More researches on this aspect are necessary to explore in the future. However, the diversity and similarity of TCR repertoire in tumor tissues and paired sentinel lymph nodes from CC patients, as well as their relationship with the prognosis of patients were analyzed in our study. Consistent with the results reported in breast cancer (37), our data showed that the TCR repertoire diversity in tumor tissues was significantly lower than in paired sentinel lymph nodes. The more restricted TCR repertoire in tumor tissue might be indicative of a specific and oligoclonal T-cell response to tumor-associated antigens that are present in and restricted to the tumor microenvironment during tumorigenesis. More interestingly, we found that less clonotypes in sentinel lymph node TCR repertoire were presented in the patients who were alive with tumor recurrence or metastasis, or had died of their disease after surgery, while more clonotypes in sentinel lymph node TCR repertoire were presented in patients who were alive with stable disease for several years (at least 2 years) after surgery. This finding is similar with the results from another study in gastric cancer, in which low diversity of adjacent normal mucosal tissue T cell repertoire predicting poor clinical prognosis has been concluded (38). Due to distinct degree of TCR repertoire overlap between tumor tissues and paired sentinel lymph nodes were observed in these CC patients (Figure 6E), we hypothesized that these overlapping clonotypes identified in sentinel lymph nodes might represent a population of effector memory T-cells that egressed from the tumor tissue, which have been reported to play an important role in post-surgery surveillance against tumor recurrence (39, 40). Of note, these findings were based on the patients with early CC who had a chance to receive a surgery before chemoradiotherapy and all the studied sentinel lymph nodes were normal lymph nodes. Therefore, further study on larger cohort is needed to determine the precise magnitude of this favorable effect, including patients with advance CC and metastatic lymph nodes.

In summary, using high-throughput sequencing we have provided a comprehensive characterization of the TCR repertoire in peripheral blood, tumor tissue and paired sentinel lymphatic node from CC patients to help us to get a better understanding of the immune response in CC patients. Although the detail immunologic mechanism remains unclear and the results presented here require further exploration, our findings suggest that circulating TCR repertoire could sever as a novel potential biomarker for immune monitoring of CC patients, and the number of clonotypes in sentinel lymphatic node TCR repertoire could predict the clinical prognosis of CC patients.

## Author contributions

J-HC: conception and design; S-HY, X-KS, and JJ: acquisition of samples; X-PC, Y-MP, and S-LM: execution of experiments; Y-BJ and X-FM: analysis of data; Y-BJ and K-RL: interpretation of data; K-RL: drafting of the manuscript; J-HC and WL: obtained funding.

### Conflict of interest statement

The authors declare that the research was conducted in the absence of any commercial or financial relationships that could be construed as a potential conflict of interest.
