# Supplementary material for: TCR Repertoire as a Novel Indicator for Immune Monitoring and Prognosis Assessment of Patients With Cervical Cancer
Source: Front Immunol. 2018 Nov 22;9:2729. doi: 10.3389/fimmu.2018.02729 (PMC6262070; doi:10.3389/fimmu.2018.02729)
Supplement: Supplementary file 1 [file Table_1.pdf]

Table S1. The clinical information and high throughput sequencing results of each peripheral blood sample from 25 CC patients, 30 CIN patients and 20 healthy women.

Groups	Sample ID	Age (years)	Clincal satge	Pathological Type	Number of V gene	Number of J gene	Number of Total TCRβ CDR3 aa sequences	Number of TCRβ CDR3 Unique aa sequences	Shannon's entropy
CC	CC1	50	IB1	Moderately differentiated adenocarcinoma	63	13	1774550	99083	11.86
	CC2	48	IB1	Moderately differentiated squamous cell carcinoma	63	13	1337082	53031	9.39
	CC3	44	IB1	Moderately and poorly differentiated squamous cell carc	63	13	1313928	33236	7.83
	CC4	50	IB1	Moderately differentiated squamous cell carcinoma	61	13	868119	36081	9.68
	CC5	54	IB1	Moderately differentiated squamous cell carcinoma	62	13	1232647	56452	11.25
	CC6	43	IB1	Moderately differentiated squamous cell carcinoma	63	13	1361985	25071	4.73
	CC7	44	IB1	Moderately differentiated adenocarcinoma	64	13	1383129	16218	6.15
	CC8	54	IB1	Moderately and poorly differentiated squamous cell carc	64	13	1357070	32359	8.77
	CC9	58	IB1	Moderately differentiated squamous cell carcinoma	62	13	1423327	32701	9.08
	CC10	38	IB1	Moderately differentiated adenocarcinoma	62	13	1421947	12487	5.71
	CC11	39	IB1	Moderately differentiated squamous cell carcinoma	60	13	932736	24506	8.07
	CC12	43	IB2	Moderately differentiated squamous cell carcinoma	62	13	731035	4895	2.25
	CC13	50	IB2	Moderately differentiated adenocarcinoma	55	13	1555579	9307	3.57
	CC14	50	IB2	Moderately differentiated adenocarcinoma & squamous	57	13	2317370	58314	8.96
	CC15	44	IB2	Moderately differentiated squamous cell carcinoma	63	13	748552	21925	7.95
	CC16	54	IIA	Poorly differentiated adenocarcinoma	61	13	1209030	16713	6.96
	CC17	54	IIB	Moderately differentiated squamous cell carcinoma	62	13	2388308	47433	8.86
	CC18	53	IIB	Moderately differentiated squamous cell carcinoma	64	13	1544705	9054	3.09
	CC19	35	IIB	Moderately differentiated squamous cell carcinoma	58	13	1434049	9584	4.38
	CC20	53	IIIB	Moderately differentiated squamous cell carcinoma	62	13	1075236	11116	5.77
	CC21	48	IIIB	Moderately differentiated squamous cell carcinoma	60	13	1645938	13704	5.42
	CC22	55	IIIB	Moderately differentiated squamous cell carcinoma	64	13	1455260	11553	4.86
	CC23	54	IVA	Moderately differentiated squamous cell carcinoma	60	13	1725250	12765	4.6
	CC24	45	IVB	Moderately differentiated squamous cell carcinoma	58	13	1010047	13210	5.75
	CC25	60	IVB	Moderately differentiated adenocarcinoma	63	13	963036	16231	5.81
	CIN1	45	CIN-1	-	61	13	1056491	41141	10.41
	CIN2	35	CIN-1	-	60	13	516789	20622	6.84
	CIN3	27	CIN-1	-	61	13	1771477	16483	5.36
	CIN4	22	CIN-1	-	65	13	1592677	34230	8.38
	CIN5	50	CIN-1	-	63	13	1521178	36462	9.36
	CIN6	50	CIN-1	-	64	13	1441418	22707	6.49
	CIN7	31	CIN-1	-	62	13	1892292	39502	7.6
	CIN8	53	CIN-1	-	61	13	1181841	12149	5.98
	CIN9	30	CIN-1	-	64	13	1832448	39961	8.43
	CIN10	31	CIN-1	-	61	13	626609	23243	9.01
	CIN11	32	CIN-2	-	60	13	676085	20435	7.31
	CIN12	34	CIN-2	-	64	13	1342912	49715	9.52
	CIN13	50	CIN-2	-	62	13	1502572	19755	6.8

CIN	CIN14	27	CIN-2	-	60	13	880097	21840	8.59
	CIN15	42	CIN-2	-	62	13	1008249	34871	10.23
	CIN16	55	CIN-2	-	63	13	1511031	42578	9.71
	CIN17	40	CIN-2	-	63	13	1554626	37338	9.16
	CIN18	33	CIN-2	-	63	13	1490492	43713	9.73
	CIN19	45	CIN-2	-	63	13	1067364	35432	9.71
	CIN20	37	CIN-3	-	62	13	1717962	20983	6.43
	CIN21	38	CIN-3	-	63	13	2023772	32055	6.38
	CIN22	43	CIN-3	-	62	13	1101932	12842	6.29
	CIN23	39	CIN-3	-	61	13	1008130	38550	9.13
	CIN24	48	CIN-3	-	62	13	1298034	32854	8.7
	CIN25	44	CIN-3	-	63	13	1858192	40902	8.54
	CIN26	43	CIN-3	-	63	13	1716479	33871	8.31
	CIN27	23	CIN-3	-	64	13	1286419	33053	8.43
	CIN28	30	CIN-3	-	56	13	629056	9094	6.41
	CIN29	29	CIN-3	-	63	13	2939431	50214	8
	CIN30	43	CIN-3	-	63	13	1867940	76503	11.03
Healthy women	Health1	34	-	-	63	13	985607	90952	11.66
	Health2	44	-	-	62	13	440549	28935	8.84
	Health3	48	-	-	64	13	678607	39764	7.8
	Health4	54	-	-	62	13	571837	48119	9.26
	Health5	59	-	-	63	13	886934	88837	12.02
	Health6	50	-	-	63	13	964906	80164	10.77
	Health7	52	-	-	62	13	842442	67712	10.77
	Health8	55	-	-	63	13	736221	62081	10.71
	Health9	53	-	-	62	13	462092	31759	8.92
	Health10	56	-	-	63	13	502974	35821	9.39
	Health11	42	-	-	64	13	886352	71703	10.4
	Health12	43	-	-	63	13	753224	86683	12.35
	Health13	45	-	-	61	13	432053	31986	7.02
	Health14	39	-	-	63	13	928187	82577	11.1
	Health15	47	-	-	63	13	744726	49315	9.68
	Health16	41	-	-	64	13	1092474	85304	10.73
	Health17	37	-	-	64	13	943125	81984	10.96
	Health18	48	-	-	64	13	1644423	50163	9.04
	Health19	54	-	-	64	13	2719021	61512	7.69
	Health20	57	-	-	62	13	752118	62181	9.74
