# Supplementary material for: TCR Repertoire as a Novel Indicator for Immune Monitoring and Prognosis Assessment of Patients With Cervical Cancer
Source: Front Immunol. 2018 Nov 22;9:2729. doi: 10.3389/fimmu.2018.02729 (PMC6262070; doi:10.3389/fimmu.2018.02729)
Supplement: Supplementary file 2 [file Table_2.pdf]

Table S2. The clinical information and high throughput sequencing results of each tumor or sentinel lymphatic node sample from 16 CC patients.

Sample ID	Age (years)	FIGO satge	Pathological Type	Disease status	Number of V gene	Number of J gene	Number of Total TCRβ CDR3 aa sequences	Number of TCRβ CDR3 Unique aa sequences	Shannon's entropy
T1	61	IB1	Moderately differentiated squamous cell carcinoma	Non-progression	60	13	2083672	38855	7.03
L1					63	13	1140369	91096	13.67
T2	67	IB1	Poorly differentiated squamous cell carcinoma	Progression (metastasis)	62	13	1438642	18222	3.88
L2					62	13	870982	85725	13.99
T3	42	IB1	Moderately differentiated squamous cell carcinoma	Progression (metastasis)	61	13	2427006	26719	4.77
L3					63	13	857880	82922	14.03
T4	54	IB1	Moderately differentiated squamous cell carcinoma	Non-progression	61	13	2098758	63083	9.32
L4					63	13	1115408	134024	14.73
T5	31	IB2	Moderately differentiated squamous cell carcinoma	Progression (dead)	63	13	2122212	51778	7.67
L5					61	13	836455	49422	12.14
T6	47	IB1	Moderately differentiated squamous cell carcinoma	Progression (metastasis)	62	13	1440264	32359	7.97
L6					61	13	791734	59630	12.93
T7	46	IB1	Moderately differentiated squamous cell carcinoma	Non-progression	62	13	1051658	33913	8.89
L7					62	13	734020	71408	14.11
T8	51	IB1	Moderately differentiated squamous cell carcinoma	Non-progression	61	13	1816074	39619	7.42
L8					63	13	1094322	89914	13.94
T9	33	IB1	Moderately differentiated squamous cell carcinoma	Unknown	63	13	1555746	43676	8.05
L9					64	13	1028897	79284	13.25
T10	28	IB1	Moderately differentiated squamous cell carcinoma	Progression (recurrence)	64	13	2779271	57394	7.19
L10					63	13	1516685	99067	13.03
T11	40	IB1	orly differentiated adenocarcinoma & squamous cell carcino	Progression (recurrence)	61	13	1571686	49259	9.68
L11					62	13	724371	81281	14.33
T12	47	IB1	Moderately differentiated squamous cell carcinoma	Non-progression	63	13	1697803	40164	7.30
L12					62	13	1074088	99905	13.78
T13	58	IB1	Moderately differentiated squamous cell carcinoma	Non-progression	62	13	2238375	62609	8.48
L13					63	13	1441663	127465	14.13
T14	54	IB1	Moderately differentiated squamous cell carcinoma	Non-progression	63	13	1970853	48302	7.71
L14					62	13	819866	114506	14.87
T15	43	IB1	loderately and poorly differentiated squamous cell carcinom	Non-progression	62	13	1459226	45600	8.19
L15					63	13	1620825	143731	14.60
T16	36	IB2	Moderately differentiated squamous cell carcinoma	progression (recurrence, metastasis, death)	64	13	2101246	31400	6.34
L16					58	13	94748	14887	7.57
