# Supplementary material for: TCR Repertoire as a Novel Indicator for Immune Monitoring and Prognosis Assessment of Patients With Cervical Cancer
Source: Front Immunol. 2018 Nov 22;9:2729. doi: 10.3389/fimmu.2018.02729 (PMC6262070; doi:10.3389/fimmu.2018.02729)
Supplement: Supplementary file 3 [file Table_3.pdf]

TABLE S3 │The detail information of disease-associated TCR clonotypes.

	CDR3 sequences	CC patients	CIN patients &Healthy	P value
**CC-associated TCR sequences**	CASSLDPGLYEQYF	9	0	0.000016
	CASSLQSSSYEQYF	9	0	0.000016
	CASSQRTSGTIYNEQFF	9	0	0.000016
	CSARDWRDYNEQFF	9	0	0.000016
	CAIRDKETRNEQFF	8	0	0.000064
	CASKAGANVLTF	8	0	0.000064
	CASSDRQTGAVSFRETQYF	8	0	0.000064
**CIN-associated TCR sequences**	**CDR3 sequences**	**CIN**	**CC patients &Healthy**	**P value**
	CASSLEQLVRSETQYF	11	0	0.000011
	CASSPGGDRLYEQYF	10	0	0.000036
	CASLQGGQETQYF	9	0	0.000114
	CASSLNGAQKETQYF	9	0	0.000114
	CASSLRQKETQYF	9	0	0.000114
	CASSPDRVADTQYF	9	0	0.000114
	CATSARDSYGYTF	9	0	0.000114
	CSAIAGSSTDTQYF	9	0	0.000114
	CSARGADWFTEQYF	9	0	0.000114
	CSARGPLVGGLGEQFF	9	0	0.000114
	CSASGGSQYF	9	0	0.000114
