# Supplementary material for: TCR Repertoire as a Novel Indicator for Immune Monitoring and Prognosis Assessment of Patients With Cervical Cancer
Source: Front Immunol. 2018 Nov 22;9:2729. doi: 10.3389/fimmu.2018.02729 (PMC6262070; doi:10.3389/fimmu.2018.02729)
Supplement: Supplementary file 4 [file Data_Sheet_1.docx]

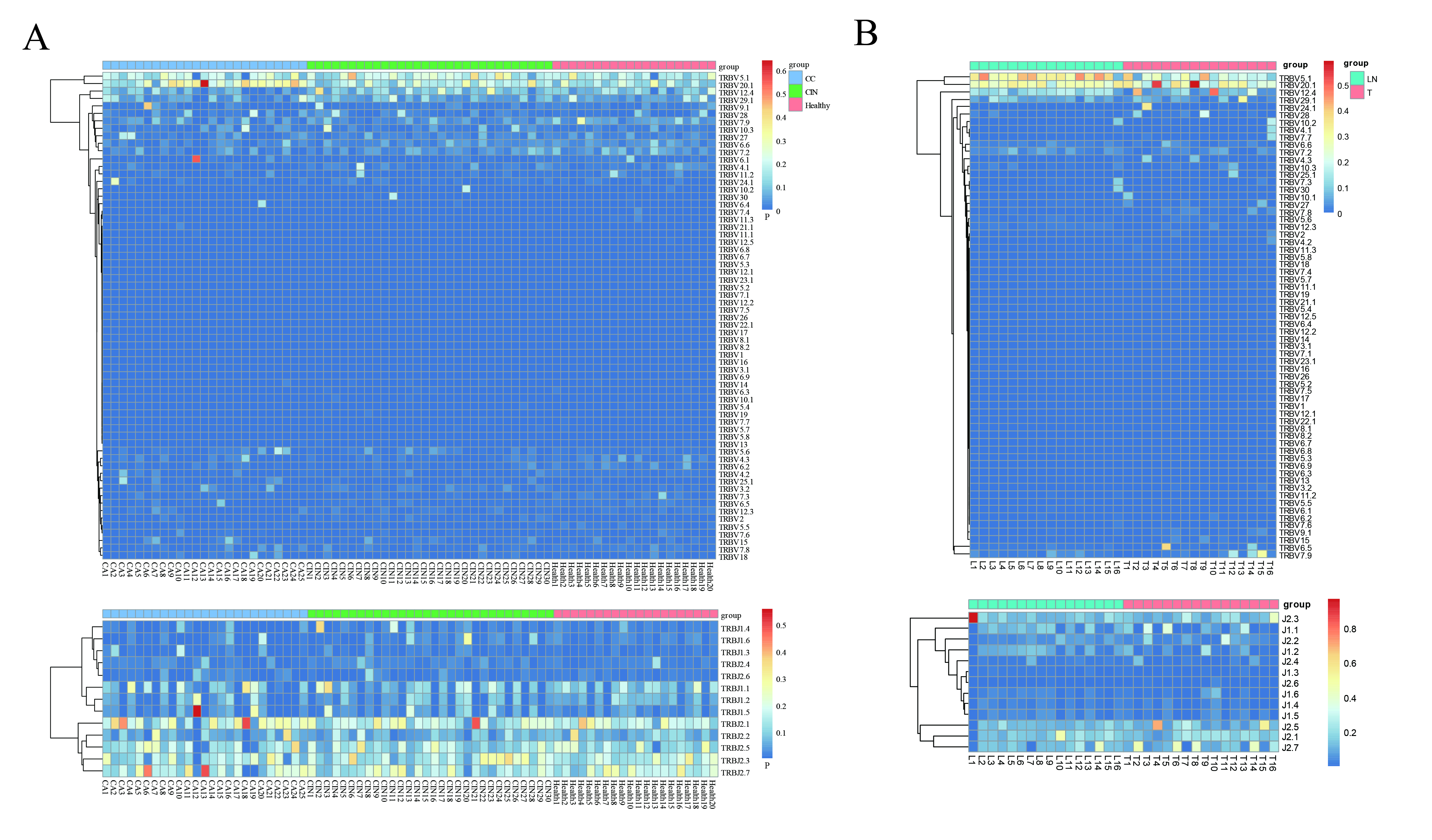


Figure S1. The heat maps of TCR β (TRB) V and J gene usage frequencies (A) in peripheral blood TCR repertoire of each sample from the CC, CIN patients and healthy women, and (B) in tumor and sentinel lymph node TCR repertoire of each sample from the CC patients.
